# HEALTH EQUITY, INTERSECTIONALITY, AND CLIMATE CHANGE: PUBLIC HEALTH CONSIDERATIONS FOR RESEARCH

**DOI:** 10.1093/geroni/igad104.1238

**Published:** 2023-12-21

**Authors:** Christina Mu, Alisha Thompson, Bailee Brekke, Patricia D’Antonio

**Affiliations:** University of South Florida, Tampa, Florida, United States; LSU, Baton Rouge, Louisiana, United States; Miami University, Oxford, Ohio, United States; The Gerontological Society of America, Washington, District of Columbia, United States

## Abstract

With the rapidly growing aging population, we are faced with an increasingly diverse population of older people. In the 2023 MedPac Report, the Commission found that health disparities by race/ethnicity and income persist. Black Medicare beneficiaries and people with low-income subsidies are at higher risk of poor health outcomes (e.g., increase hospitalization, readmission, and emergency department visits) compared to non-Hispanic White, Asian, and higher income beneficiaries. In the coming years, the aging population will be faced with novel challenges that threaten individuals’ pursuit of health, well-being, and life. The focus of this presentation will be to discuss three emerging research areas including health disparities, intersectionality, and climate change. All are distinctly important and interconnected with health research more broadly. As a GSA summer policy intern, I researched legislation and synthesized literature related to these topics. For instance, I monitored and reviewed the Pandemic All Hazards Preparedness Act, Survival Aid for Emergencies Through Medicare Act, and MedPac recommendations to promote health equity. Additionally, I reviewed and contributed to GSA sign-on letters for congressional legislation. Within my own research focused on the joint associations of sleep and pain, this internship experience has provided new insights in line with my dissertation, which also seeks to uncover differences by age, sex, and race/ethnicity, as well as understand how contextual factors may influence the individual’s health. Looking forward, researchers may have vested interested in contributing to gaps in knowledge regarding health disparities and climate change.

